# Unique Electron-Transfer-Mediated Electrochemiluminescence of AuPt Bimetallic Nanoclusters and the Application in Cancer Immunoassay

**DOI:** 10.3390/bios13050550

**Published:** 2023-05-16

**Authors:** Huiwen Zhou, Ruanshan Liu, Guangxing Pan, Miaomiao Cao, Ling Zhang

**Affiliations:** 1 School of Science, Harbin Institute of Technology, Shenzhen 518055, China; 21s058046@stu.hit.edu.cn (H.Z.); 22s058051@stu.hit.edu.cn (R.L.); 2 Shenzhen Key Laboratory of Flexible Printed Electronics Technology, Harbin Institute of Technology, Shenzhen 518055, China; guangxingpan@stu.hit.edu.cn (G.P.); caomm12@yeah.net (M.C.); 3 School of Materials Science and Engineering, Harbin Institute of Technology, Shenzhen 518055, China

**Keywords:** ECL, AuPt nanoclusters, alpha fetoprotein, immunoassay, cancer diagnosis

## Abstract

Noble Metal nanoclusters (NCs) are promising electrochemiluminescence (ECL) emitters due to their amazing optical properties and excellent biocompatibility. They have been widely used in the detection of ions, pollutant molecules, biomolecules, etc. Herein, we found that glutathione-capped AuPt bimetallic NCs (GSH-AuPt NCs) emitted strong anodic ECL signals with triethylamine as co-reactants which had no fluorescence (FL) response. Due to the synergistic effect of bimetallic structures, the ECL signals of AuPt NCs were 6.8 and 94 times higher than those of monometallic Au and Pt NCs, respectively. The electric and optical properties of GSH-AuPt NCs differed from those of Au and Pt NCs completely. An electron-transfer mediated ECL mechanism was proposed. The excited electrons may be neutralized by Pt(II) in GSH-Pt and GSH-AuPt NCs, resulting in the vanished FL. Furthermore, abundant TEA radicals formed on the anode contributed electrons to the highest unoccupied molecular orbital of GSH-Au_2.5_Pt NCs and Pt(II), booming intense ECL signals. Because of the ligand effect and ensemble effect, bimetallic AuPt NCs exhibited much stronger ECL than GSH-Au NCs. A sandwich-type immunoassay for alpha fetoprotein (AFP) cancer biomarkers was fabricated with GSH-AuPt NCs as signal tags, which displayed a wide linear range from 0.01 to 1000 ng·mL^−1^ and a limit of detection (LOD) down to 1.0 pg·mL^−1^ at 3S/N. Compared to previous ECL AFP immunoassays, this method not only had a wider linear range but also a lower LOD. The recoveries of AFP in human serum were around 108%, providing a wonderful strategy for fast, sensitive, and accurate cancer diagnosis.

## 1. Introduction

Au and the relevant nanoclusters (NCs) have been widely applied in sensing, catalysis, and bioimaging due to their unique quantum-confined electronic effects, optical, biocompatibility, and eco-friendly properties [1,2,3]. The small size of Au nanoclusters (below 4 nm) makes them have ‘molecule-like’ properties [4]. Generally, Au NCs were formed under the protection of ligands by strong adsorptions. On the one hand, Au NCs were usually synthesized in the organic phase, and alkanethiolate [5,6], adamantanethiol [7], and phosphine [8,9] were applied as the ligands and strong reducing agents such as NaBH_4_, B_2_H_6_, and H_2_ were used. The structures including the number of Au atoms, ligands, and shapes of Au NCs were exactly designed. On the other hand, it was feasible to synthesize Au NCs in the aqueous phase with water-soluble molecules such as thiolates with carboxyl, amino acids, proteins, and ds DNA as ligands. The produced water-soluble Au NCs processed distinctive biocompatibility, optics, and catalytic activities, enabling Au NCs applied in immunoassays, and the detections of heavy metal ions, dyes and amino acid. For example, GSH protected AuPt bimetallic NCs (GSH-AuPt NCs) had horse radish peroxidase mimicking properties which were applied in the detection of H_2_O_2_ and further the colorimetric point-of-care testing of glucose by naked eyes with linear range of 5–55 µM [10]. Bovine serum albumin-protected Au NCs (BSA-Au NCs) exhibited two fluorescence (FL) emission peaks around 430 and 610 nm under the excitation wavelength of 360 nm, which was decreased by malachite green due to the FL resonance energy transfer [11]. Thus, 0.3~20 μM malachite green was detected with a low limit of detection (LOD) of 0.19 μM. Furthermore, Chen et al. developed methods to prepare 2.4 nm methionine-capped Au NCs (Met-Au NCs) owing to intense orange FL signals at 530 nm [12]. Cu^2+^ and Co^2+^ selectively annealed the FL of Met-Au NCs through coordination interactions between Cu^2+^ and Met ligands [12,13]. With FL test paper, 1 μM of Cu^2+^ or Co^2+^ can be detected by bare eyes under 365 nm UV excitations. Ag-doped 5-mercapto-1-tetrazolea-acetic acid sodium salt (MTAS) capped Au NCs increased the FL, which was applied in the detection of cysteine selectively in the linear range of 0.05–25 µM due to the annealing effect [14]. Carcinoembryonic antigen (CEA) and AFP were applied as the model proteins in the FL immunoassay with Au NCs and CdTe quantum dots (QDs) coated Si nanospheres, leading the linear detection range of 0.1–400 ng·mL^−1^ for both CEA and AFP [15].

Electrochemiluminescence (ECL) has been widely applied in analysis for the distinguishing features of fast, ultra-sensitive, and miniaturized analysis, which are superior to FL and colorimetric methods somehow. The ECL properties of Au-relevant NCs and their applications in analysis have been studied a lot because of their advantages in wide detection range, low background signal, facile production, biocompatibility, eco-friendliness, and so on [16,17,18]. Xu et al. found that 11-mercaptoundecanoic acid-capped Au NCs (MUA-Au NCs) with surface defects promoted monochromatic anodic ECL with N_2_H_4_ as the co-reactant [19]. A broad linear detection range of 5 × 10^−4^–1.0 U·mL^−1^ with a LOD of 0.1 mU·mL^−1^ for CA125 cancer biomarkers was obtained [19]. Weng et al. investigated the ECL quenching effect of Cu^2+^ on pre-oxidation-treated Met-Au NCs, reaching the ultra-low detection range of 1.0 × 10^−18^–1.0 × 10^−14^ M for Cu^2+^ and a low LOD of 2.3 × 10^−20^ mol·L^−1^ without any other amplification technique [20]. Additionally, it was found that BSA had a significant quenching effect on the ECL signal of N-acetyl-L-cysteine capped Au NCs, enabling the construction of a highly sensitive and simple ECL sensing platform for BSA (LOD, 3.2 × 10^−10^ mol·L^−1^) [21]. Moreover, Met-Au NCs were demonstrated to emit ECL in the near-infrared region (NIR) with triethanolamine (TEOA) as the co-reactant, which had been applied as signal tags in the alpha-fetoprotein (AFP) immunoassay [22]. A wide linear range of 3 × 10^−15^–1 × 10^−10^ g·mL^−1^ with a LOD of 1 × 10^−15^ g·mL^−1^ for AFP was achieved [22].

Although the ECL assay of Au NCs has been fabricated, the ECL efficiency is necessary to be enhanced to obtain a better analytic performance. In recent years, continuous efforts have been made to improve the ECL efficiency of Au NCs. The ligands of Au NCs affected the properties a lot. For example, Met-Au NCs showed enhanced ECL by 75 times compared with BSA-Au NCs [22]. Additionally, the modifications of capping agents of Au NCs enabled direct ECL emission in the absence of co-reactants [23,24,25,26]. Besides, bimetallic Au-related NCs exhibited stronger ECL than monometallic Au NCs due to the electronic effect (ligand effect) and the geometrical effect (ensemble effect) [27,28,29]. Fu et al. found that ECL in NIR of Met-Au NCs can be enhanced after doping Ag, which lowered the LOD of CA125 by one order compared with MUA-Au NCs in an ECL immunoassay [19,30]. Jia et al. designed an ion doping strategy by doping Co^2+^ to cysteamine and N-acetyl-L-cysteine stabilized Au NCs, which reduced the surface defects of NCs and promoted electron transfer due to the synergistic effect of Au and Co^2+^ [31]. As a result, the ECL performances of the Au NCs were improved which can be applied in the detections of neuron specific enolase in the linear range of 0.5 fg·mL^−1^ng·mL^−1^ [31].

As a cancer biomarker, AFP is classified as an oncofetal glycoprotein, having a molecular weight of 70 kDa approximately [32,33]. It is mainly formed in the liver, yolk sac, and the gastrointestinal tract of the human fetus [34]. Neonatal serum AFP content is generally 10–50 μg·mL^−1^ and gradually decreases with growth [35]. AFP is not produced in mature and healthy hepatocytes, and serum AFP levels in healthy adults are below 20 ng·mL^−1^ [36]. However, when people suffer from certain malignant tumors, AFP concentrations in serum are usually higher than 25 ng·mL^−1^ [37,38,39]. Therefore, it is very important to develop new methods to detect AFP in human serum accurately, which is of great significance for the early diagnosis of diseases.

In this regard, we found that the ECL efficiency of GSH-AuPt bimetallic NCs was enhanced by 6.8 times compared with GSH-Au NCs due to the fast electron transfer. The ECL onset potential of GSH-AuPt NCs was 90 mV in advance of GSH-Au NCs. The electrons were easier to be transferred from triethylamine (co-reactant, TEA) radicals to the lowest unoccupied molecular orbital (LUMO) of AuPt NCs at the oxidation states on the anode, which turned into excited states. Then electrons in LUMO of AuPt NCs returned to the highest occupied molecular orbital (HOMO) to form ground-state AuPt NCs, emitting photons at the same time. Moreover, there were ligand and ensemble effects for AuPt bimetallic NCs, leading to much stronger ECL than GSH-Au NCs. There was no FL response for GSH-AuPt or GSH-Pt NCs, and there were ignored ECL responses for GSH-Pt NCs. It was found that the Pt(II) valence state dominated in GSH-AuPt and GSH-Pt NCs according to XPS results. It suggested that the electrons of LUMO would be combined with Pt(II) after the excitations by photons, thus, there were no excited GSH-AuPt or GSH-Pt NCs formed. Moreover, there no FL was emitted correspondingly. Similarly, most electrons from TEA radicals were neutralized by Pt(II) in GSH-Pt NCs, leading to an ultra-weak ECL. Thanks to the unique ECL properties of GSH-AuPt NCs, a sandwich immunoassay for AFP with a wide linear detection range was fabricated successfully. The linear detection range for AFP was 0.01–1000 ng·mL^−1^ (LOD = 1.0 pg·mL^−1^ at 3 S/N).

## 2. Materials and Methods

### 2.1. Reagents

HAuCl_4_·4H_2_O (BC grade), H_2_PtCl_6_·6H_2_O (Reagent grade), NaOH (Reagent grade), KH_2_PO_4_ (BC grade), K_2_HPO_4_ (ACS grade), K_4_Fe(CN)_6_·3H_2_O (Biochemical grade), trisodium citrate dihydrate (Reagent grade), n-hexadecyltrimethylammonium chloride (Reagent grade, CTAC), NaCl (Reagent grade), and KCl (Molecular biology grade) were purchased from Sangon Biotech (Shanghai) Co., Ltd. (Shanghai, China) Reduced L-glutathione (Reagent grade, GSH), bovine serum albumin (Reagent grade, BSA), K_3_Fe(CN)_6_ (Reagent grade), n-hydroxysuccinimide (Reagent grade, NHS), and streptavidin (Biotech grade) were obtained from Sigma-Aldrich. N, N-diisopropylethylamine (DIPEA, 0.75 g·mL^−1^, Reagent grade), H_2_O_2_(≥30%, Analytical reagent), trimethylamine (TMA, 0.63 g·mL^−1^, Reagent grade), 2-(dimethylamino) ethanol (DMEA, 0.89 g·mL^−1^, Reagent grade), 2-(dibutylamino) ethanol (DBAE, 0.86 g·mL^−1^, Reagent grade), triethanolamine (TEOA, 1.12 g·mL^−1^, Analytical reagent), tripropylamine (TPrA, 0.75 g·mL^−1^, Chemically pure), diethylamine (DEA, 0.71 g·mL^−1^, Analytical reagent), 3-(3-dimethylaminopropyl)-1-ethylcarbodiimide hydrochloride (EDC·HCl, Reagent grade), sodium borohydride (98%), H_2_SO_4_ (98 wt%, Reagent grade), HCl (36–38 wt%, Reagent grade), HNO_3_ (65–68 wt%, Reagent grade) and isopropyl alcohol (IPA, 0.79 g·mL^−1^, Reagent grade) were obtained from Tansole Co., Ltd. (Shanghai, China). Triethylamine (TEA, 0.73 g·mL^−1^, ≥99.5%) was obtained from Aladdin. N_2_H_4_·H_2_O (>98%) was obtained from Innochem (Beijing) Technology Co., Ltd. (Beijing, China) Normal human serum were purchased from Beijing Solarbio Technology Co., Ltd. (Beijing, China) Human alpha fetoprotein antigen (AFP Ag, 1 mg·mL^−1^), biotinylated AFP monoclonal antibody (AFP biotin-Ab_1_, 1 mg·mL^−1^), AFP monoclonal antibody (AFP Ab_2_, 3.4 mg·mL^−1^), human prostate specific antigen (PSA, 2.0 mg·mL^−1^), human chorionic gonadotropin beta (beta-hCG, 2.74 mg·mL^−1^, containing 0.05% NaN_3_), human carcinoembryonic antigen (CEA, 1.0 mg·mL^−1^, containing 0.05% NaN_3_), and recombinant human cytokeratin K19 (Cyfra211, 3.0 mg·mL^−1^, containing 0.2% NaN_3_) were obtained from Linc-Bio Science Co., Ltd. (Shanghai, China). Phosphate buffer solution (PBS) at pH 7.4 was prepared by mixing KH_2_PO_4_ and K_2_HPO_4_ solutions. 100 mg·mL^−1^ EDC and 100 mg·mL^−1^ NHS aqueous solutions were prepared in water, respectively. TEA, DIPEA, TMA, TEOA, DMEA, DBAE, TPrA, DEA, N_2_H_4_·H_2_O, and H_2_O_2_ solutions were prepared in 0.1 mol·L^−1^ PBS at pH 7.4 (PBS1) for electrochemical tests, respectively. 10 mg·mL^−1^ BSA and 1 mg·mL^−1^ streptavidin aqueous solution were both prepared in 10 mM PBS at pH 7.4 containing 0.15 M NaCl (PBS2). AFP Ag, AFP biotin-Ab_1_, AFP Ab_2_, PSA, beta hCG, and CEA solutions were dispersed in PBS2 as purchased. Cyfra211 were dispersed in Tris-HCl solutions at about PH 7.4 as received. All the chemicals were used without further purification. Milli-Q water (18.2 MΩ cm^−1^) was used throughout the experiments. The glassware used in the synthesis of metal NCs was soaked in the fresh HNO_3_/HCl (1:3, *v*/*v*) solution for about 30 min, rinsed thoroughly with ultrapure water, and dried subsequently before use.

### 2.2. Synthesis of GSH-AuPt NCs

GSH-capped AuPt (GSH-AuPt) NCs were prepared according to the previous report with a minor modification [10]. The mole ratio of HAuCl_4_:H_2_PtCl_6_ during the synthesis was adjusted to optimize the ECL properties of GSH-AuPt NCs. Typically, 5 mL of HAuCl_4_ (20 mmol·L^−1^) was completely mixed with 5 mL H_2_PtCl_6_ of different concentrations (20, 10, 6.67, 5, and 4 mmol·L^−1^), and then the mixed solution was slowly introduced into GSH solution (15 mmol·L^−1^, 10 mL). The final solution was stirred at 90 °C for 6.5 h. GSH-AuPt NCs crude products were obtained by adding 25 mL IPA to the above solutions and washed twice by centrifugations at 12,000 rpm for 5 min with IPA. Then GSH-AuPt NCs were dried at 37 °C in an oven for 4 h. The resulting powders were stored at 4 °C.

GSH-Au NCs and GSH-Pt NCs were synthesized by the same procedure without H_2_PtCl_6_ or HAuCl_4_ as precursors added. 5 nm Au nanoparticles were synthesized by the previous method [40]. Briefly, 0.5 mL of 5 mmol·L^−1^ HAuCl_4_ and 0.6 mL of 10 mmol·L^−1^ sodium borohydride solutions were subsequently injected into 10 mL of 100 mmol·L^−1^ CTAC aqueous solution under vigorous stirring. Then the solutions were stored for 1 day at room temperature in the dark. Finally, the solutions were centrifugated at 12,000 rpm for 10 min and the products were washed with water by centrifugation three times. 10 nm Au nanoparticles were synthesized by reducing HAuCl_4_ with citrate. Briefly, 1.5 mL of 10 mmol·L^−1^ trisodium citrate solution was quickly added into the 10 mL of 0.5 mmol·L^−1^ boiled HAuCl_4_ solutions under refluxing. The solutions were heated for 30 min, and the products were collected and washed by centrifugations.

### 2.3. Fabrications of Ab_2_-GSH-AuPt NCs

Ab_2_ coating GSH-AuPt NCs (Ab_2_-GSH-AuPt NCs) were prepared through acylation reactions. First, 5 mg GSH-AuPt NCs dissolved in 5 mL PBS2 were added to 100 µL of 100 mg·mL^−1^ EDC and 100 µL of 100 mg·mL^−1^ NHS solutions and stirred at room temperature for 30 min to activate the carboxyl groups of GSH. Then, GSH-AuPt NCs were purified with IPA and washed by centrifugations. The resulting paste was re-dissolved in 4 mL PBS2, then slowly added with 1 mL of 100 μg·mL^−1^ AFP Ab_2_ and stirred at room temperature for 6.5 h. The final Ab_2_-AuPt NCs products were purified with IPA and washed by centrifugations. Finally, the resulting paste was re-dissolved in 4.5 mL PBS2 and slowly added with 0.5 mL of 10 mg·mL^−1^ BSA aqueous solutions, then stirred at room temperature for 1 h. The final products were purified with IPA and washed by centrifugations. The resulting paste was dissolved in 5 mL PBS2 and stored at 4 °C for future use.

### 2.4. Fabrications of ECL Sandwich Immunoassay

**Scheme 1:**
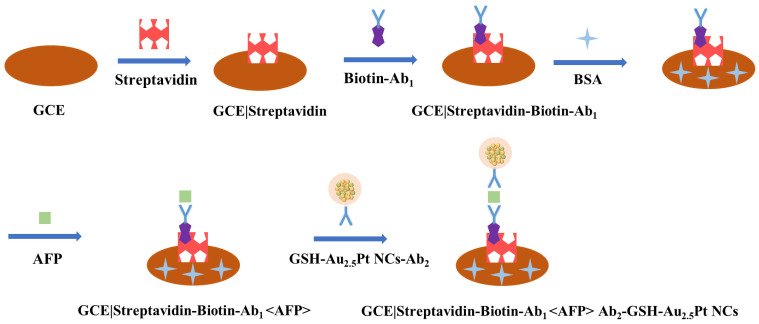
Schematic illustrations for fabricating ECL immunosensor of AFP.

The ECL immunoassay was fabricated on glassy carbon electrodes (GCEs) via sandwich-type structures (Scheme 1). Before modifications, GCEs with diameters of 5 mm were polished with 0.3 and 0.05 μm Al_2_O_3_ powders successively and rinsed thoroughly with Milli-Q water and dried in a N_2_ stream. After that, 20 μL of 1 mg·mL^−1^ streptavidin solution was dropped onto GCEs to be incubated for 2 h at room temperature. Then GCEs were rinsed in PBS2 three times to fabricate GCE|Streptavidin. Further, 20 μL of 1 mg·mL^−1^ biotin-Ab_1_ solutions were dropped onto GCEs and incubated for 2 h at room temperature. Then GCEs were rinsed in PBS2 three times to fabricate GCE|Streptavidin-Biotin-Ab_1_. After that, 20 µL of 10 mg·mL^−1^ BSA solutions were dropped onto GCEs and incubated at room temperature for 30 min to block non-specific binding sites. Then GCE|Streptavidin−Biotin-Ab_1_ was further linked with the AFP target antigen via dropping 20 μL of AFP solution at different concentrations onto the GCE surface and incubated for 2 h, then rinsed with PBS2 three times to fabricate GCE|Streptavidin−Biotin-Ab_1_<AFP>. The obtained GCE|Streptavidin−Biotin-Ab_1_ <AFP> were incubated with 20 μL Ab_2_-GSH-AuPt NCs solutions for 2 h to form GCE|Streptavidin−Biotin-Ab_1_<AFP>Ab_2_-GSH-AuPt NCs sandwich structures. ECL signals were generated by applying potentials from 0 to 1.2 V at 50 mV·s^−1^ in PBS1 containing 0.2 mol·L^−1^ TEA.

### 2.5. Electrochemical Measurements

The related electrochemical measurements were obtained on a CHI 760E electrochemical analyzer (CH Instruments, Chenhua Co., Ltd. Shanghai, China). The related ECL measurements were obtained on an MPI-AII ECL analyzer (Xi’an Remex Analytical Instrument Co., Ltd., Xi’an, China). Three-electrode systems with Ag/AgCl (saturated KCl) as reference electrodes, platinum pieces as counter electrodes, and modified GCEs as working electrodes were used in all electrochemical and ECL measurements. Electrochemical impedance spectra (EIS) of modified GCEs were measured at open circuit potentials (0.223 V) in the range of 100 kHz~10 Hz with a sinusoidal potential perturbation of 5 mV.

### 2.6. Instruments

UV–vis absorption spectroscopy was conducted on the U-2910 spectrophotometer. Fluorescence (FL) spectra were recorded with an FL-970 spectrophotometer. Transmission electron microscopy (TEM) characterizations, high-resolution TEM (HRTEM) images, high-angle annular dark-field scanning TEM (HAADF-STEM) images, and energy dispersive spectroscopy (EDS) for elemental mapping analysis was taken on JEOL JEM-3200FS field-emission microscope operated at 300 kV. X-ray diffraction (XRD) patterns were collected on Rigaku SmartLab (Cu Kα radiation). X-ray photoelectron spectroscopy (XPS) was recorded with PHI VersaProbe 4. Fourier transform infrared (FT-IR) spectra were collected on Nicolet is50 with KBr pellets (Thermo Fisher Scientific, Waltham, MA, USA).

## 3. Results and Discussion

### 3.1. Synthesis and Characterizations of GSH-AuPt NCs

**Figure 1:**
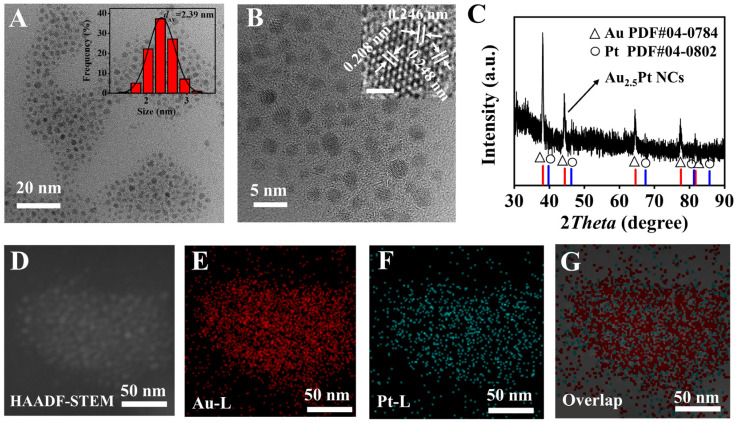
TEM image (**A**), HRTEM image (**B**), XRD pattern (**C**), HAADF-STEM (**D**), and EDS images (**E**–**G**) of GSH-Au_2.5_Pt NCs. Inset of (**A**), size distribution curve of GSH-Au_2.5_Pt NCs. Inset of (**B**), HRTEM, scale bar, 1 nm.

**Figure 2:**
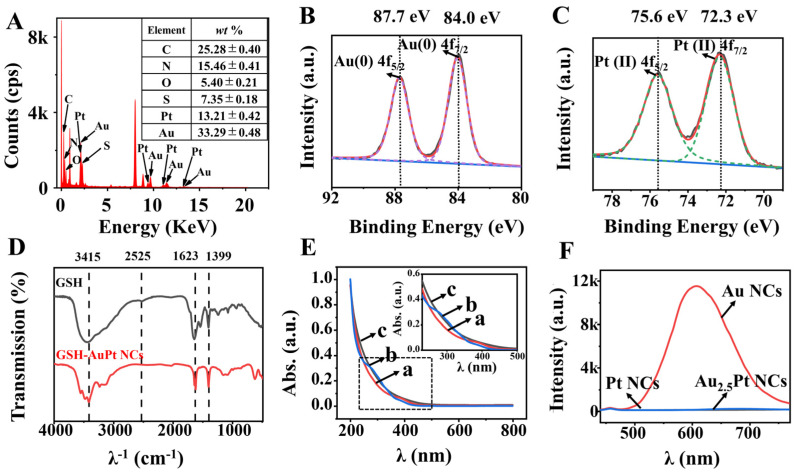
EDS spectrum (**A**), XPS spectra for Au 4f (**B**) and Pt 4f (**C**) of GSH-Au_2.5_Pt NCs. Inset of (**A**), mass percentages of elements in GSH-Au_2.5_Pt NCs. (**D**) FT-IR spectra of GSH and GSH-Au_2.5_Pt NCs powders. (**E**) UV-vis absorbance spectra of GSH-Au NCs (a), GSH-Pt NCs (b), and GSH-Au_2.5_Pt NCs (c) in water, respectively. (**F**) FL spectra of three NCs in water. Excitations, 396 nm.

GSH-AuPt NCs were synthesized by reducing HAuCl_4_ and H_2_PtCl_6_ precursors with GSH at 90 °C for 6.5 h in aqueous solutions [10]. The mole ratio of Au and Pt precursors introduced during synthesis was optimized to achieve the maximum ECL activity. When the mole ratio of HAuCl_4_:H_2_PtCl_6_ was 3:1, the obtained atomic ratio of Au:Pt for AuPt NCs was 2.5:1 according to TEM-EDS analysis (named as Au_2.5_Pt, Figure 1 and Figure 2A). A good mono-dispersity in size was obtained for GSH-Au_2.5_Pt NCs, which were measured to be 2.39 ± 0.31 nm according to TEM characterizations (Figure 1A). The HRTEM image (Figure 1B) showed that the lattice spacings of GSH-Au_2.5_Pt along three directions were 0.246, 0.248, and 0.208 nm, which were closed to (111), (111), and (100) lattice of face-centered cubic (fcc) Au and Pt crystals [41,42,43]. The XRD pattern of GSH-Au_2.5_Pt NCs exhibited a set of peaks with 2θ values of 38.16°, 44.35°, 64.53°, 77.53° and 81.73°, demonstrating the formations of alloyed structures of Au_2.5_Pt NCs (Figure 1C). Because the content of Au in alloyed Au_2.5_Pt NCs was greater than that of Pt, the diffraction angles of Au_2.5_Pt NCs were more closed to those of fcc Au (JCPDS 04-0784) [30,42,44,45]. According to HAADF-STEM and EDS analysis, Au and Pt atoms were uniformly distributed in as-prepared Au_2.5_Pt NCs, confirming Au_2.5_Pt alloy nanostructures were obtained (Figure 1D–G). Au_2.5_Pt NCs were encapsulated by GSH, and the mass percentages of N, S, Au, and Pt elements were 15.46%, 7.35%, 33.29%, and 13.21% by TEM-EDS analysis, respectively (Figure 2A).

The surface compositions and elemental valence states of GSH-Au_2.5_Pt NCs were identified by XPS (Figure 2 and Figure S2). The signals of N, S, Au, and Pt elements were exhibited, which were consistent with EDS characterizations. The high-resolution XPS spectra of Au 4f and Pt 4f bands were shown in Figure 2B,C. The two characteristic peaks around 84.0 and 87.7 eV demonstrated the existence of Au (0) within GSH-Au_2.5_Pt NCs [46,47,48,49]. Two characteristic peaks around 72.3 and 75.6 eV appearing in the XPS spectrum of Pt 4f indicated Pt (II) valence dominated within GSH-Au_2.5_Pt NCs [50,51,52]. It suggested that the sulfydryl groups of GSH tend to interact with Pt atoms of Au_2.5_Pt NCs, leading to the dominant presence of Pt(II) and Au(0). FT-IR transmission spectra of as-prepared GSH-Au_2.5_Pt NCs were further conducted to analyze the surface molecules adsorbed (Figure 2D). The spectra of Au_2.5_Pt NCs were similar to those of GSH ligands, indicating Au_2.5_Pt NCs were successfully protected by external GSH ligands. The FT-IR transmittance peak at 1399 cm^−1^ fits the stretching vibrations of the C−O bonds. The stretching vibration expansion of C=O bonds resulted in a characteristic peak at 1623 cm^−1^ [53]. In addition, the characteristic peak for stretching vibrations of O−H bonds was found at 3415 cm^−1^. The stretching vibration of S-H bonds appeared at 2525 cm^−1^ in the FT-IR spectra of GSH, as shown in Figure 2D. The signals for S-H bonds were much lower than those of O-H stretching and C=O stretching expansion vibrations. For GSH-Au_2.5_Pt NCs, the stretching vibration peak of S-H shifted to the lower wavenumber (2430 cm^−1^), which should be attributed to the formation of S-Pt bonds [54]. Due to the effective capping and protecting effects of GSH, the obtained GSH-Au_2.5_Pt NCs showed good homogeneity and water solubility, rather than aggregating into large particles.

The monometallic Au (*d*, 2.50 ± 0.29 nm) and Pt (*d*, 1.93 ± 0.19 nm) NCs were also formed without adding Pt or Au precursors, respectively (Figure S1). The lattice spacing of 0.239 and 0.235 nm fit to (111) facets of Au and Pt NCs, respectively. Moreover, the characteristic elements, N and S for GSH encapsulated on Au and Pt NCs were also shown in XPS spectra (Figure S2B,C). In the XPS of Au 4f of pure GSH-Au NCs, it was observed that the binding energy was shifted higher for 0.2 eV compared to GSH-Au_2.5_Pt, suggesting the presence of Au(I) valence state (Figure S2D). More importantly, the binding energy of Pt 4f for pure GSH-Pt NCs was the same as GSH-Au_2.5_Pt, further confirming that Pt atoms in bimetallic NCs tend to be oxidized first when surrounded by GSH capping agents (Figure S2E).

For the UV-vis properties, GSH-Au_2.5_Pt NCs had little absorptions in the range of 200–800 nm (Figure 2E, curve c). In contrast, GSH-Au NCs had an absorption peak of around 396 nm and GSH-Pt NCs had two absorption peaks of around 300 and 393 nm, respectively (Figure 2E, curves a and b). For the FL properties, there were no photons emitting for GSH-Au_2.5_Pt or Pt NCs after the excitations at 396 nm (Figure 2F). It can be seen that GSH-Au NCs showed strong FL emissions at 604 nm. It indicated that Pt had annealing effects for FL of Au NC, which may be due to the electrons in HOMO of Au atoms being transferred into Pt(II) of Au_2.5_Pt NCs [1,28,55]. For Pt NCs, the electrons in HOMO were hard to excite or jump to LUMO, thus, there were no FL signals. The FL intensity of the Au NCs disperser decreased after adding Pt NCs, also suggesting the annealing effects of Pt (Figure S3A).

Furthermore, the special FL properties were only owned by Au NCs which were like molecules due to their ultra small size (below 4 nm). When the sizes of Au nanomaterials increased to 5 and 10 nm, the FL signals disappeared. Moreover, there were strong absorptions around 550 nm in the UV-vis spectra (Figures S4 and S5).

### 3.2. ECL Properties of GSH-Au_2.5_Pt NCs

**Figure 3:**
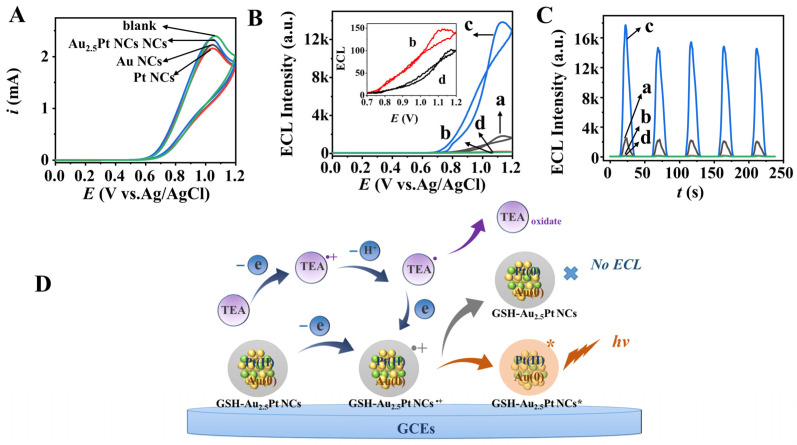
CVs (**A**), ECL-potential profiles (**B**), and ECL-time profiles (**C**) of 1 mg·mL^−1^ GSH-Au (a), GSH-Pt (b), GSH-Au_2.5_Pt (c) NCs and blank (d) solutions containing 0.2 M TEA in PBS1. Scanning rates, 50 mV·s^−1^; PMT, –700 V. Inset of (**B**), enlarged ECL-potential curves for GSH-Pt NCs (b) and blank (d). (**D**) ECL mechanism of GSH-Au_2.5_Pt NCs with TEA as the co-reactant.

The anodic ECL of Au_2.5_Pt NCs in the presence of co-reactants, i.e., TEA was investigated by cyclic voltammetry (CV) methods (Figure 3). It was shown that there were strong oxidation peaks corresponding to oxidations of TEA around 1.06 V (Figure 3A) [56,57]. After introducing GSH Au_2.5_Pt, GSH-Au, and GSH-Pt NCs, the peak currents for TEA decreased a little, which may be due to the increased solution resistance and slower diffusion rates of TEA. The onset potential of the GSH-Au_2.5_Pt NCs/TEA system was 0.523 V vs. Ag/AgCl, which was in advance of 13 mV to GSH-Pt NCs/TEA and 16 mV to GSH-Au NCs/TEA system. It suggested that GSH-Au_2.5_Pt NCs had electrochemically catalytic activity to the oxidation of TEA. Correspondingly, ECL began to emerge when the potentials scanned to 0.653, 0.743, and 0.764 V for GSH-Au_2.5_Pt, GSH-Au, and GSH-Pt NCs (Figure 3B). The largest ECL intensity was formed during the backward scans. The peaked ECL intensity of the GSH-Au_2.5_Pt NCs/TEA system was 6.8 and 94 times higher than those of GSH-Au NCs/TEA and GSH-Pt NCs/TEA, respectively (Figure 3C). Moreover, the ECL intensity of the GSH-Pt NCs/TEA and TEA systems were too small to be ignored. Thus, we can conclude that GSH-Au_2.5_Pt NCs process the catalytic activity to ECL reactions, due to the electrochemically generated photons emissions and the presence of abundant co-reactants. It was very different from the FL phenomena. Both Au and Pt play an important role in the formation of strong ECL, and synergistic effects were present. ECL intensity of GSH-Au NCs solutions was not increased after mixing with varied amounts of GSH-Pt NCs, further confirming the super ECL performance of Au_2.5_Pt bimetallic NCs (Figure S3B,C). Further, there were no ECL signals for 5 or 10 nm Au nanoparticles (Figure S4C,D). Moreover, the FL and ECL properties were mainly generated by Au and AuPt NCs, which were not dependent on the kinds of ligands [22,23,24,25,26].

On the anode, TEA was oxidized first to form positively charged TEA^•+^ radical cations, which were further deprotonated to form neutral TEA^•^ radicals (Equations (1) and (2), Figure 3D) [58,59]. Then GSH-Au_2.5_Pt NCs were oxidized into GSH-Au_2.5_Pt NCs^•+^ radical cations at a higher potential, which can gain one electron from TEA^•^ radicals and turn into excited states (Equations (3) and (4)). In this process, there would be two kinds of combination paths for the gained electrons. A part of the electrons from TEA^•^ were neutralized by Pt(II) of GSH-Au_2.5_Pt NCs^•+^ (Figure 3D). Moreover, the left occupied the LUMO of GSH-Au_2.5_Pt NCs^•+^ to form GSH-Au_2.5_Pt NCs* and ECL (Equation (5)). Thanks to the presence of Au, the second path dominated for GSH-Au_2.5_Pt NCs, resulting in a much stronger ECL than those of pure GSH-Pt NCs. The ligand and ensemble effects of bimetallic GSH-AuPt NCs enabled much stronger ECL than monometallic GSH-Au NCs.

TEA − e^−^ = TEA^•+^ (1)

TEA^•+^ − H^+^ = TEA^•^ (2)

GSH-Au_2.5_Pt NCs − e^−^ = GSH-Au_2.5_Pt NCs^•+^ (3)

GSH-Au_2.5_Pt NCs^•+^ + TEA^•^ = GSH-Au_2.5_Pt NCs* + TEA_oxidate_ (4)

GSH-Au_2.5_Pt NCs* = GSH-Au_2.5_Pt NCs + *hν* (5)

**Figure 4:**
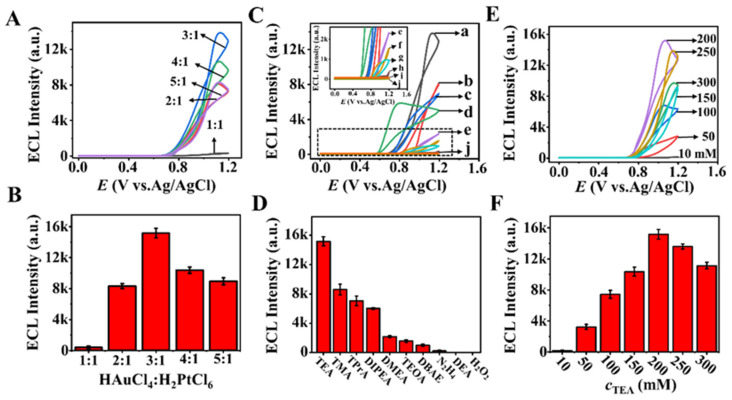
Effects of mole ratios of HAuCl_4_ to H_2_PtCl_6_ in the preparations of NCs (**A**,**B**), co-reactants (a) TEA; (b) TMA; (c) TPrA; (d) DIPEA; (e) DMEA; (f)TEOA; (g) DBAE; (h) N_2_H_4_; (i) DEA; (j) H_2_O_2_ (**C**,**D**), and concentrations of TEA (**E**,**F**) to ECL signals of 1 mg·mL^−1^ GSH-Au_2.5_Pt NCs in PBS1. Scanning rates, 50 mV·s^−1^; Co-reactants, 0.2 mol·L^−1^; PMT, –700 V.

**Figure 5:**
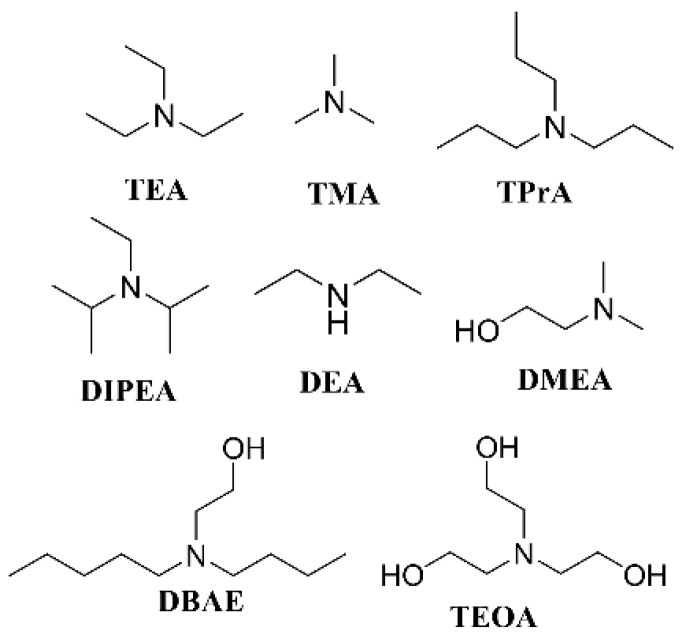
Molecule structures of different co-reactants in Figure 4D.

The contents of Au and Pt of GSH-Au_2.5_Pt NCs affected the ECL obviously (Figure 4A,B). With the increased amount of H_2_PtCl_6_ precursors in the preparations, the ECL intensity increased and reached the maximum when the mole ratio of HAuCl_4_:HAuCl_6_ was 3:1. Too much Pt in NCs decreased the ECL signals, fitting to the above results shown in Figure 3. Furthermore, the types of co-reactants affected the ECL signals of GSH-Au_2.5_Pt NCs a lot (Figure 4C,D). The tertiary amine co-reactants promoted ECL, such as TEA, TMA, TPrA, and DIPEA (Figure 5). Too small or big alkyl groups in tertiary amines did not benefit the ECL of NCs. Moreover, hydroxy groups inhibited the ECL of GSH-Au_2.5_Pt NCs, and the ECL of NCs in the presence of DMEA, TEOA, and DBAE were very low. N_2_H_4_, secondary amine (DEA), and H_2_O_2_ cannot induce the ECL of GSH-Au_2.5_Pt NCs. It was proposed that there were no radicals produced during the oxidation process of the molecules with hydroxy groups with high reducibility (Figure S6). Moreover, DEA and H_2_O_2_ were hard to oxidize on anodes. In addition, it was found that the concentration of TEA affected the ECL intensity of GSH-Au_2.5_Pt NCs largely (Figure 4E,F). With the increase in the TEA concentration, the ECL intensity of the system increased dramatically first (0.01–0.2 mol·L^−1^ TEA) and then decreased slowly (0.2–0.3 mol·L^−1^ TEA). Sufficient TEA was important to achieve the higher ECL of GSH-Au_2.5_Pt NCs, and 0.2 mol·L^−1^ was the optimized concentration.

Notably, the ECL onset potential of GSH-Au_2.5_Pt NCs with DIPEA as the co-reactant was in advance of 120 mV to the TEA system. The onset oxidation potential of DIPEA was in advance of 25 mV to the TEA system. Thus, a different ECL mechanism would be present for GSH-Au_2.5_Pt NCs in the presence of DIPEA. First, DPIEA was oxidized on the anode to be DPIEA^•+^ radical cations, which were more oxidative and can oxidize GSH-Au_2.5_Pt NCs into GSH-Au_2.5_Pt NCs^•+^ (Equations (6) and (7)). Then, DPIEA^•+^ was deprotonated into DPIEA^•^, which reduced GSH-Au_2.5_Pt NCs^•+^ into GSH-Au_2.5_Pt NCs*, generating ECL emissions (Equations (5), (8) and (9)).

DIPEA − e^−^ = DIPEA^•+^ (6)

DIPEA^•+^ + GSH-Au_2.5_Pt NCs = DIPEA + GSH-Au_2.5_Pt NCs^•+^ (7)

DIPEA^•+^ − H^+^ = DIPEA^•^ (8)

DIPEA^•^ + GSH-Au_2.5_Pt NCs^•+^ = GSH-Au_2.5_Pt NCs* + DIPEA_oxidate_ (9)

### 3.3. ECL Performance of AFP Immunoassay

**Figure 6:**
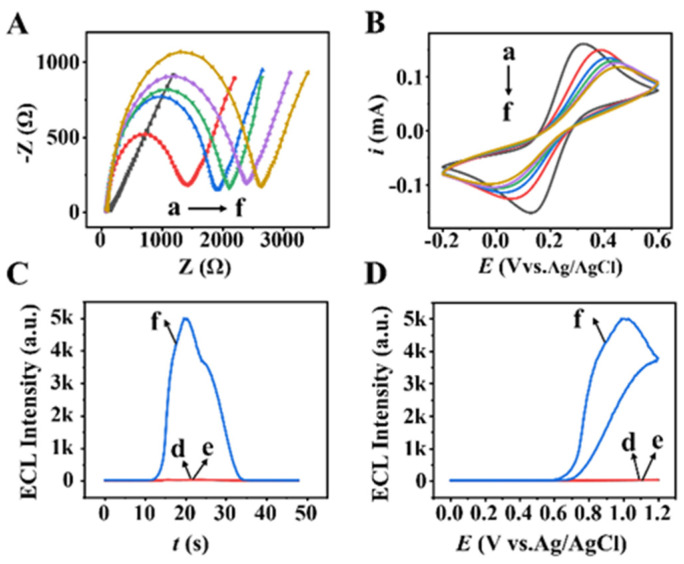
EIS curves (**A**) and CV curves (**B**) of GCE (a), GCE|Streptavidin (b), GCE|Streptavidin-Biotin-Ab_1_ (c), GCE|Streptavidin-Biotin-Ab_1_-BSA (d), GCE|Streptavidin-Biotin-Ab_1_<AFP> (e), and GCE|Streptavidin-Biotin Ab_1_<AFP>Ab_2_-GSH-Au_2.5_Pt NCs (f) in 0.1 M KCl solutions containing 5 mmol·L^−1^ K_3_Fe(CN)_6_ and K_4_Fe(CN)_6_, respectively. ECL-time (**C**) and ECL-potential (**D**) profiles of electrodes d, e, and f in PBS1 containing 0.2 mol·L^−1^ TEA. Scanning rates, 50 mV·s^−1^; PMT, −700 V.

With GSH-Au_2.5_Pt NCs as ECL signal tags, we successfully constructed a sandwich immunosensor and realized the trace detection of AFP (Scheme 1 and Part 2.4). The formations of GCE|Streptavidin-Biotin-Ab_1_<AFP>Ab_2_-GSH-Au_2.5_Pt NCs were demonstrated by EIS (Figure 6A). The resistance of the double layer on electrodes controlled by electron transfer kinetics (*R*_ct_, Ω) was reflected by the arc diameter at the low-frequency zone. As the control, bare GCE exhibited the smallest *R*_ct_ (Figure 6A, curve a), and largest redox peak currents of Fe(CN)_6_^3–^/Fe(CN)_6_^2–^ electronic pair in CVs correspondingly (Figure 6B, curve a). With the layers of streptavidin, biotin-Ab_1_, BSA, AFP, and Ab_2_-GSH Au_2.5_Pt NCs stacking onto GCEs gradually, *R*_ct_ was increased from 127 to 2667 Ω and the redox peak currents in CVs decreased accompanying the enlarged peak potential differences between anode and cathode (∆*E*, V) (Table 1). It indicated the successful fabrications of AFP ECL immunosensors. Further, strong ECL signals were observed in TEA solutions after Ab_2_-GSH-Au_2.5_Pt NCs tags were adsorbed on GCE through specific immune binding between Ab_2_ and AFP (Figure 6C,D).

### 3.4. Detections of AFP and Real Sample Analysis

**Figure 7:**
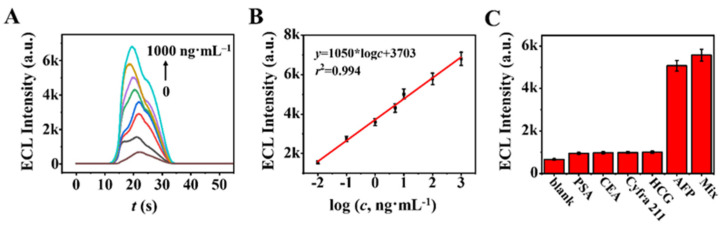
(**A**) ECL intensities of the immunosensors in 0.1 M PBS1 containing 0.2 mol·L^−1^ TEA at 50 mV·s^−1^ to detect 0, 0.01, 0.1, 1, 5, 10, 100, and 1000 ng·mL^−1^ AFP, respectively. PMT, –800 V. (**B**) Calibration curve for AFP detections. (**C**) ECL intensities of the immunosensors towards blank, 100 ng·mL^−1^ PSA, CEA, Cyfra 211, and HCG, and 10 ng·mL^−1^ AFP, and the mixtures of them.

Under the optimal conditions, we realized the trace detection of AFP by using GCE|Streptavidin-Biotin Ab_1_<AFP>Ab_2_-GSH-Au_2.5_Pt NCs ECL immunosensors. With the increase in AFP concentration, the ECL signal increased gradually (Figure 7A). Moreover, the ECL intensities were increased slowly when the concentrations of AFP were higher than 100 ng·mL^−1^ (Figure S7). The plot of ECL intensity versus logarithmic of AFP concentration displayed linearity from 0.01 to 1000 ng·mL^−1^ (*r*^2^ = 0.994) with LOD as 1.0 pg·mL^−1^ (S/N=3) (Figure 7B). Compared with the previous reports for AFP ECL immunosensors, the linear range of our method was wider (Table 2). Moreover, the sensitivity of the AFP immunosensor with GSH-Au_2.5_Pt ECL tags was better than those with Cu NCs, QDs, and Ru complex. Furthermore, the synthesized GSH-Au_2.5_Pt NCs had the advantages of simple synthesis, low biological toxicity, and high stability, meeting the requirements of detecting AFP for actual tumor patients (such as *c*_AFP_ > 500 ng·mL^−1^ for hepatocellular carcinoma in serum [33]). A similar detection range was also obtained with the ECL immunosensor with SiO_2_ nanoparticles carrying carbon QDs and Au nanoparticles as tags and molecularly imprinted polymer as AFP capturing materials (0.001–1000 ng·mL^−1^) [60]. The ECL immunosensor in this work exhibited superior anti-interfering ability (Figure 7C). A series of proteins, i.e., PSA, CEA, Cyfra211, PSA, and beta-HCG with the concentration 10 times of AFP targets did not show ECL signals. Moreover, the mixtures of all the proteins showed the same ECL intensity as pure AFP samples. It was clear that the selectivity of the immunosensor was mainly determined by the specific interaction between Ab and Ag, and there was no non-specific adsorption of Ab_2_-GSH Au_2.5_Pt NCs onto GCEs [61,62].

**Figure 8:**
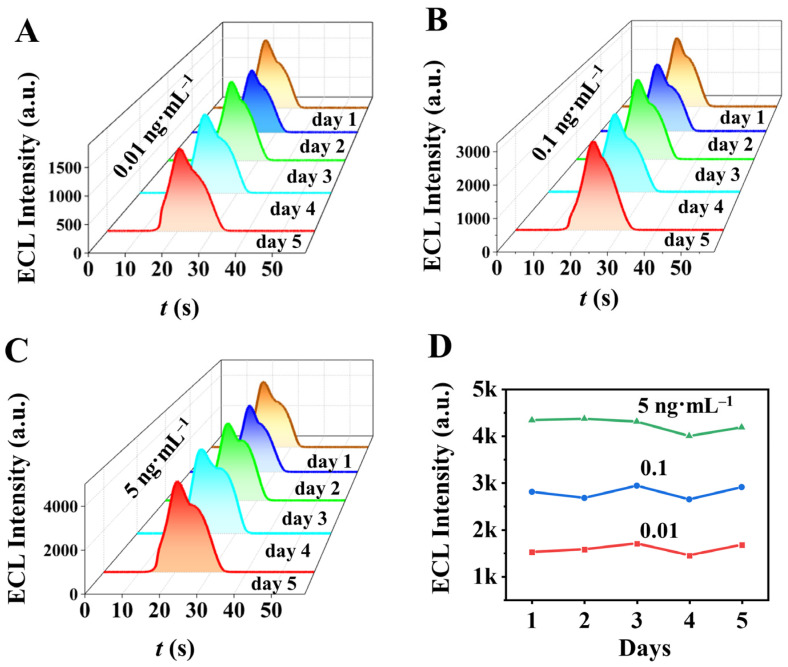
Reproducibility and stability of the fabricated immunosensors applied in detecting 0.01 (**A**), 0.1 (**B**), and 5 (**C**) ng·mL^−1^ AFP, respectively. (**D**) Peaked ECL intensities in ECL-time profiles for the five days. Scanning rates, 50 mV·s^−1^; TEA, 0.2 mol·L^−1^; PMT, −800 V.

Storage stability and reproducibility were important factors in evaluating the performance of an immunosensor. To explore them, five immunosensors were fabricated in parallel to be used for detecting one AFP sample in five days, respectively (a new immunosensor was used every day, and the others were stored at 4 °C). Three samples with 0.01, 0.1 and 5 ng·mL^−1^ AFP were tried, respectively. As shown in Figure 8, the reproducibility and stability of the ECL immunosensors was very good, the standard deviations for the five immunosensors were lower than 5.5% for all three samples. Compared with the commercial enzyme-linked immunosorbent assay (ELISA) from Sangon Biotech (Shanghai) Co., Ltd. (Shanghai, China), our method had advantages in sensitivity and detection range. The detection range for commercial ELISA is 1.56–100 ng·mL^−1^ with a sensitivity of 0.94 g·mL^−1^. The coefficient of variations for ELISA was smaller than 10% for the 20 samples tested on the same plate or different ones. In our method, the standard deviations of five electrodes for one sample were smaller than 5.5%. Thus, the accuracy and reliability of our method is comparable to commercial ELISA (Figure 8D). 

By detecting AFP in normal human serum samples, the potential application of the proposed immunosensor in practical samples was verified. The human serum samples achieved contained various chemical components, such as plasma proteins, peptides, fats, carbohydrates, growth factors, hormones, salts, and so on. Fresh serum was diluted 100 times with PBS2 before use. Then, different concentrations of AFP (1, 6, 60, and 350 ng·mL^−1^) were added to the real samples to be tested according to standard addition methods (Table 3) [75,76,77]. The recoveries were 116.80%, 107.30%, 105.22% and 104.39%, while corresponding RSDs were 3.50%, 2.31%, 2.73% and 2.11%, respectively, confirming the accuracy and sensitivity of the constructed immunosensor.

## 4. Conclusions

Above all, we have found the unique ECL properties of GSH-Au_2.5_Pt NCs which have been applied in the fast, sensitive, and accurate detections of AFP cancer biomarkers as signal tags. The ECL responses were promoted by the synergistic effects of bimetallic NCs, bringing much stronger intensities than those of pure GSH-Au and GSH-Pt NCs. This work studied the ligand and assembled effects of bimetallic NCs to ECL properties, revealing a new ECL mechanism of Au-relevant alloy NCs. It is very significant to improve the performance of ECL immunosensors for cancer diagnosis. Further, the point of care analysis is necessary for personal health care and convenient diagnosis of diseases. The ECL intensities of AuPt NCs should be enhanced so that the common camera and mobile phone could catch the signals. The strategies could be engineering the surface structures of AuPt NCs with defects and doping, and the coordinative environments of AuPt NCs with ligands, SiO_2_ nanoparticles, and metal-organic frameworks.

## Figures and Tables

**Table 1:** *R*_ct_ (Ω) and ∆*E* (V) of GCEs after layer-by-layer assembling in Figure 5.

GCEs	*R*_ct_	∆*E*
a	127	0.195
b	1400	0.332
c	1906	0.390
d	2122	0.430
e	2401	0.461
f	2667	0.483

**Table 2:** Comparisons of analytical performances of AFP ECL immunosensor in this work to the previous reports.

ECL Tags	Co-Reactant	Linear Range (ng·mL^−1^)	LOD (pg·mL^−1^)	Ref.
Cu NCs	K_2_S_2_O_8_	1–400	20.1	[63]
rGO@Au@Ru-SiO_2_	BNQDs	0.0001–100	0.03	[64]
Au-g-C_3_N_4_ NHs	K_2_S_2_O_8_	0.001–5	0.5	[65]
Au@Zn_2_SnO_4_	K_2_S_2_O_8_	0.001–50	3.4	[66]
Ru(II)@SiO_2_	TPrA	0.002–50	1.8	[67]
Cu_2_O@OMC-Ru	TPrA	0.0001–10	0.01	[68]
N-CQDs	K_2_S_2_O_8_	0.01–100	3.3	[69]
CdSe QDs	K_2_S_2_O_8_	0.05–200	2.0	[70]
CdTe NCs	DBAE	0.01–80	5.0	[59]
Ru(phen)_3_^2+^	TPrA	0.05–20	6.2	[71]
Ru(bpy)_3_^2+^	TPrA	0.05–50	40	[72]
CdTe QDs	K_2_S_2_O_8_	1–200	320	[73]
CdSe	H_2_O_2_	0.002–32	2	[74]
GSH-AuPt_2.5_ NCs	TEA	0.01–1000	1.0	This work

**Table 3:** Recoveries of the fabricated AFP ECL immunosensors.

Added (ng·mL^−1^)	Detected (ng·mL^−1^)	Recovery (%)	RSD (%)
1	1.21, 1.12, 1.18, 1.20, 1.13	116.80	3.50
6	6.38, 6.25, 6.66, 6.47, 6.43	107.30	2.31
60	61.44, 63.02, 61.53, 65.41, 64.27	105.22	2.73
350	353.67, 363.42, 374.61, 361.67, 366.43	104.39	2.11

## Data Availability

Not applicable.

## Supplementary Materials

The following supporting information can be downloaded at: https://www.mdpi.com/article/10.3390/bios13050550/s1, Figures S1–S7. Figure S1, TEM (A) and HRTEM (B) images of GSH-Au NCs. TEM (C) and HRTEM (D) images of GSH-Pt NCs. Inset of (A and C), size distributions. Figure S2, XPS spectra of GSH-Au_2.5_Pt (A), GSH-Au (B), and GSH-Pt (C) NCs. XPS spectra of Au 4f of GSH-Au (D), and Pt 4f of GSH-Pt (E) NCs. Figure S3, (A) FL spectra of GSH-Au NCs, GSH-Pt NCs, GSH-Au_2.5_Pt NCs, and the mixtures of GSH-Au NCs and GSH-Pt NCs with mass ratio of 1:2. (B and C) ECL intensity-potential profiles and peaked intensity of GSH-Au NCs (a), GSH-Pt NCs (b), GSH-Au_2.5_Pt NCs (c), the mixture of GSH-Au and GSH-Pt NCs in a mass ratio of 1:1 (d), 3:1 (e), 5:1(f), and blank solution (g), respectively. Electrolytes, 0.1 M PBS containing 0.2 M TEA; Scanning rates, 50 mV·s^−1^; PMT, –700 V. Figure S4, UV-vis absorption spectra and FL spectra of 5 nm (A) and 10 nm (B) Au nanoparticles. (A); ECL-time (C) and ECL-potential profiles (D) of 1 mg·mL^−1^ 5 nm Au nanoparticles (a), 10 nm Au nanoparticles (b), and blank (c) solutions containing 0.2 M TEA in PBS1. Scanning rates, 50 mV·s ^−1^; PMT, –700 V. Figure S5, SEM image of 10 nm Au nanoparticles. Figure S6, CVs of different co-reactants in the presence of GSH-Au_2.5_Pt NCs in 0.1 M PBS. Scanning rates, 50 mV·s^−1^; Co-reactants, 0.2 M. Figure S7, Peaked ECL intensities in the ECL-*t* profiles versus concentrations of AFP. 0.1 M PBS1 containing 0.2 mol·L^−1^ TEA at 50 mV·s^−1^; PMT, –800 V.
