# Supplementary material for: Unique Electron-Transfer-Mediated Electrochemiluminescence of AuPt Bimetallic Nanoclusters and the Application in Cancer Immunoassay
Source: Biosensors (Basel). 2023 May 16;13(5):550. doi: 10.3390/bios13050550 (PMC10216212; doi:10.3390/bios13050550)
Supplement: Supplementary file 1 [file biosensors-13-00550-s001.zip › biosensors-2311149-supplementary.pdf]

biosensors

MDPI

Supporting Information

Unique Electron-Transfer-Mediated Electrochemiluminescence of AuPt Bimetallic Nanoclusters and the Application in Cancer Immunoassay

Huiwen Zhou [1,2], Ruanshan Liu [1,2], Guangxing Pan [2,3], Miaomiao Cao [2,3] and Ling Zhang [1,2,*]

[1] School of Science, Harbin Institute of Technology, Shenzhen 518055, China; 21s058046@stu.hit.edu.cn (H.Z.); 22s058051@stu.hit.edu.cn (R.L.)
[2] Shenzhen Key Laboratory of Flexible Printed Electronics Technology, Harbin Institute of Technology, Shenzhen 518055, China; guangxingpan@stu.hit.edu.cn (G.P.); caomm12@yeah.net (M.C.)
[3] School of Materials Science and Engineering, Harbin Institute of Technology, Shenzhen 518055, China
* Correspondence: zhangling2018@hit.edu.cn

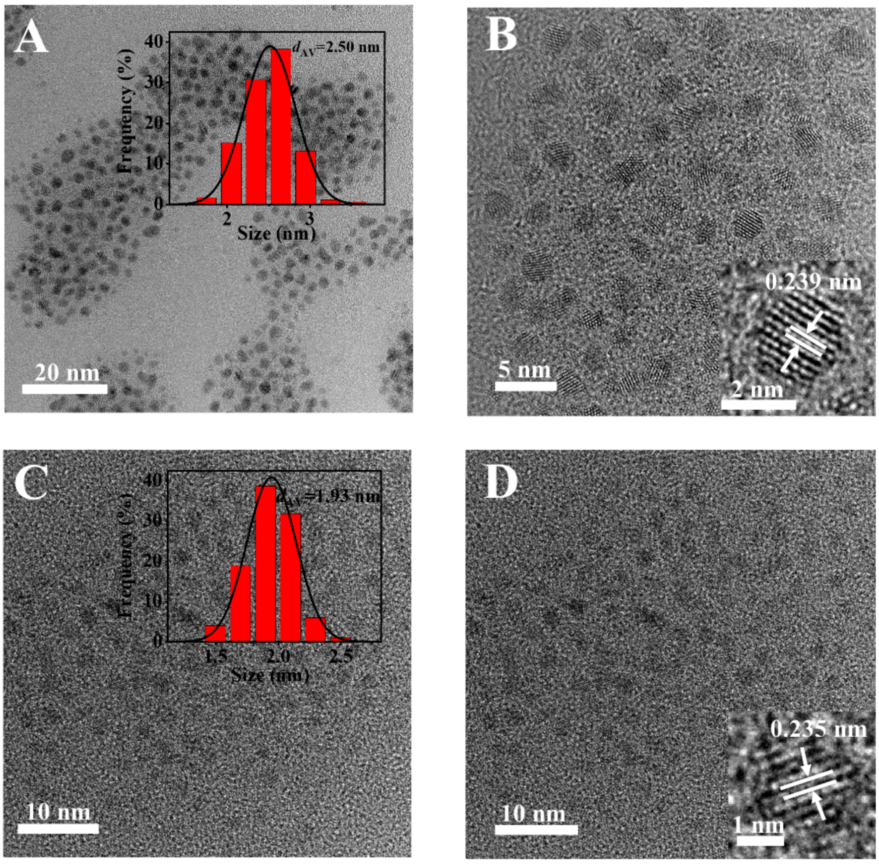

Figure S1. TEM (**A**) and HRTEM (**B**) images of GSH-Au NCs. TEM (**C**) and HRTEM (**D**) images of GSH-Pt NCs. Inset of (A and C), size distributions.

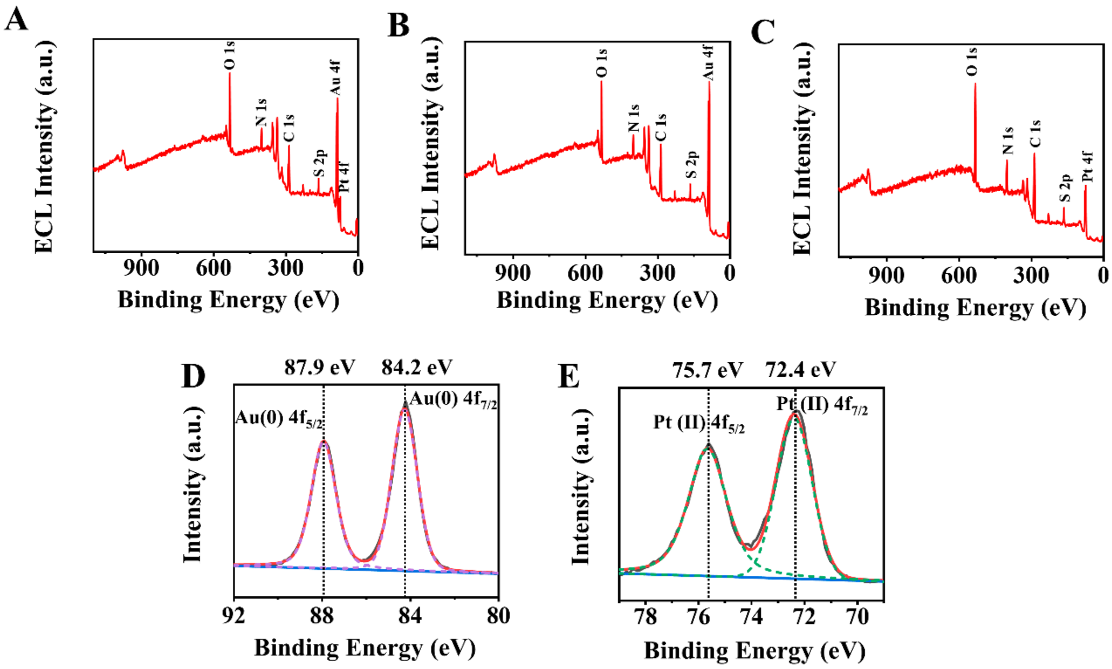

Figure S2. XPS spectra of GSH-Au2.5Pt (**A**), GSH-Au (**B**), and GSH-Pt (**C**) NCs. XPS spectra of Au 4f of GSH-Au (**D**), and Pt 4f of GSH-Pt (**E**) NCs.

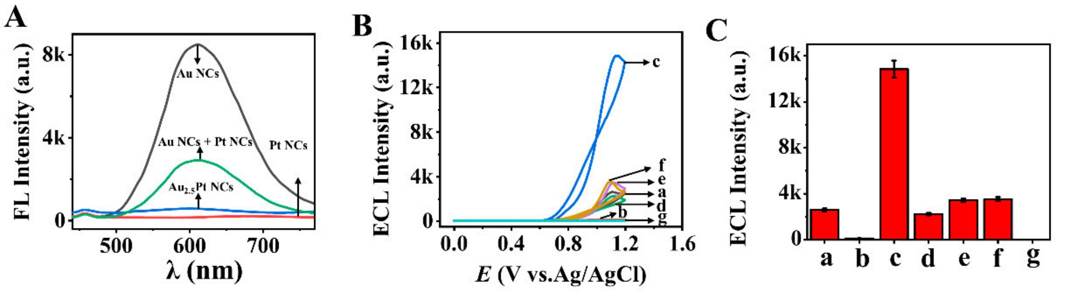

Figure S3. (**A**) FL spectra of GSH-Au NCs, GSH-Pt NCs, GSH-Au2.5Pt NCs, and the mixtures of GSH-Au NCs and GSH-Pt NCs with mass ratio of 1:2. (**B** and **C**) ECL intensity-potential profiles and peaked intensity of GSH-Au NCs (a), GSH-Pt NCs (b), GSH-Au2.5Pt NCs (c), the mixture of GSH-Au and GSH-Pt NCs in a mass ratio of 1:1 (d), 3:1 (e), 5:1(f), and blank solution (g), respectively. Electrolytes, 0.1 M PBS containing 0.2 M TEA; Scanning rates, 50 mV·s^{-1}; PMT, –700 V.

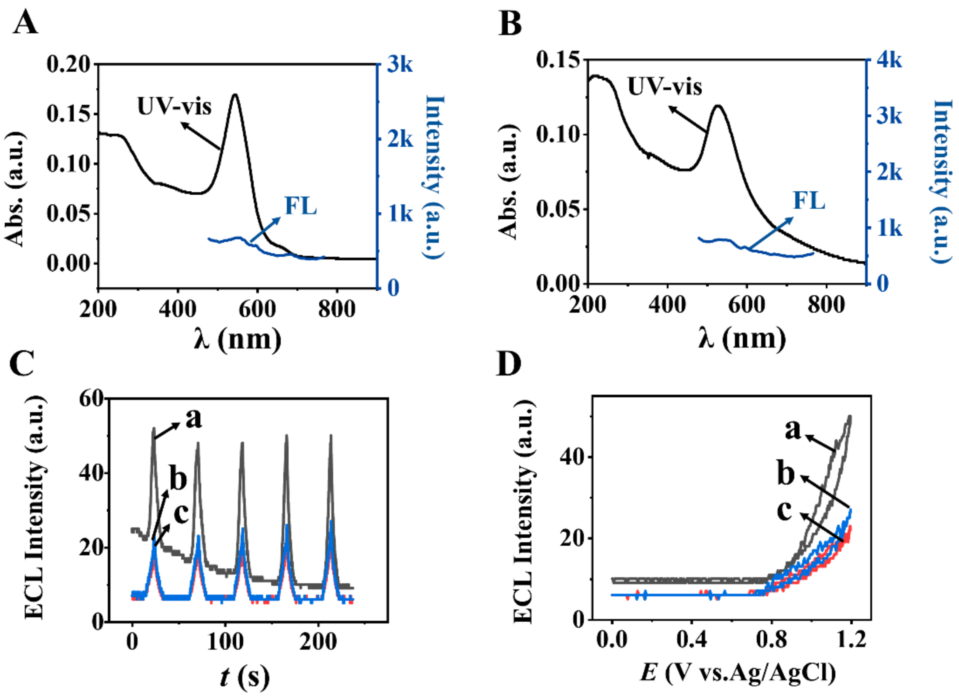

Figure S4. UV-vis absorption spectra and FL spectra of 5 nm (**A**) and 10 nm (**B**) Au nanoparticles. (A); ECL-time (**C**) and ECL-potential profiles (**D**) of 1 mg·mL^{-1} 5 nm Au nanoparticles (a), 10 nm Au nanoparticles (b), and blank (c) solutions containing 0.2 M TEA in PBS1. Scanning rates, 50 mV·s $^{-1}$; PMT, –700 V.

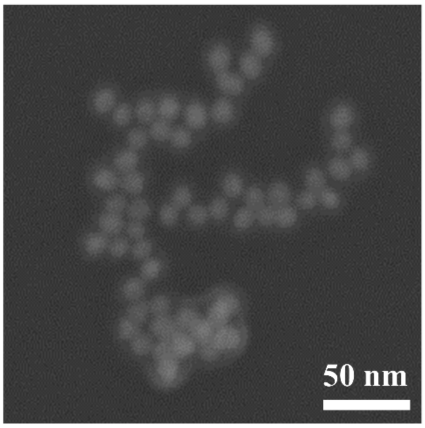

Figure S5. SEM image of 10 nm Au nanoparticles.

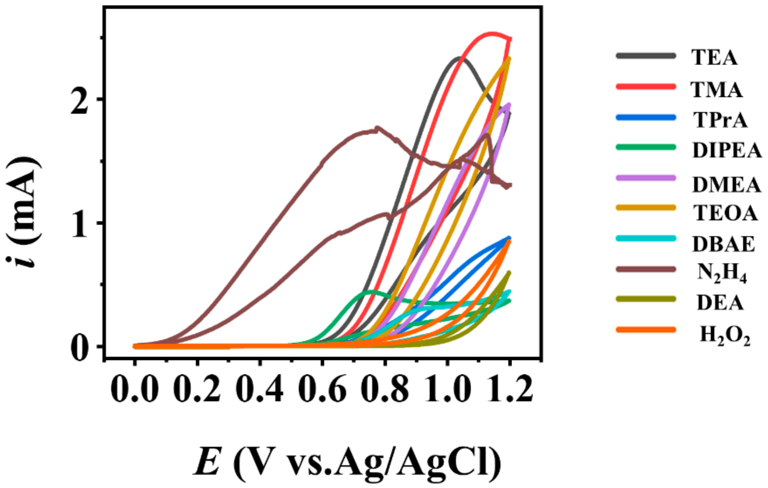

Figure S6. CVs of different co-reactants in the presence of GSH-Au2.5Pt NCs in 0.1 M PBS. Scanning rates, 50 mV·s⁻¹; Co-reactants, 0.2 M.

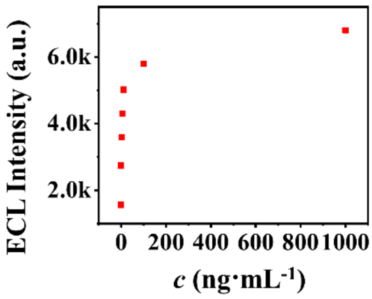

Figure S7. Peaked ECL intensities in the ECL-*t* profiles versus concentrations of AFP. 0.1 M PBS1 containing 0.2 mol·L⁻¹ TEA at 50 mV·s⁻¹; PMT, –800 V.
